# Effectiveness and cost-effectiveness of ozone treatment in patients with paraesthesia (numbness, tingling) secondary to chemotherapy-induced peripheral neuropathy: randomized, triple-blind clinical trial (OzoParQT)

**DOI:** 10.1186/s12885-025-15399-9

**Published:** 2026-01-22

**Authors:** Bernardino Clavo, Angeles Cánovas-Molina, Sara Cazorla-Rivero, Gregorio Martínez-Sánchez, Saray Galván, Gretel Benítez, Mario Federico, Himar Fabelo, Jesús M. González-Martín, Miguel A. García-Bello, Elba Lago-Moreno, Carla Antonilli, Avinash Ramchandani, Juan A. Díaz-Garrido, Minerva Navarro, Ruth Martín-Alfaro, Haidé Hernández-López, Gustavo M. Callico, Francisco Rodríguez-Esparragón

**Affiliations:** 1 Research Unit, Hospital Universitario de Gran Canaria Dr. Negrín, Las Palmas de Gran Canaria, 35019 Spain; 2 Chronic Pain Unit, Dr. Negrín University Hospital, Las Palmas de Gran Canaria, 35019 Spain; 3 Radiation Oncology Department, Hospital Universitario Dr. Negrín, Las Palmas de Gran Canaria, 35019 Spain; 4 Fundación Canaria Instituto de Investigación Sanitaria de Canarias (FIISC), Las Palmas de Gran Canaria/Tenerife, Spain; 5 University Institute for Research in Biomedicine and Health (iUIBS), Molecular and Translational Pharmacology Group, University of Las Palmas de Gran Canaria, Las Palmas de Gran Canaria, 35016 Spain; 6 Instituto Universitario de Enfermedades Tropicales y Salud Pública de Canarias de la Universidad de La Laguna, La Laguna, Tenerife 38296 Spain; 7 CIBER de Enfermedades Infecciosas, Instituto de Salud Carlos III, Madrid, 28029 Spain; 8 Spanish Group of Clinical Research in Radiation Oncology (GICOR), Madrid, 28290 Spain; 9 Scientific Advisor, Freelance, Ancona, 60126 Italy; 10 Medical Oncology Department, Hospital Universitario de Gran Canaria Dr. Negrín, Las Palmas de Gran Canaria, 35019 Spain; 11 Medical Oncology Department, Complejo Hospitalario Universitario Insular Materno-Infantil de Gran Canaria, Las Palmas de Gran Canaria, 35016 Spain; 12 Network for Research on Chronicity, Primary Care, and Health Promotion (RICAPPS), Barcelona, 08007 Spain; 13 Servicio de Evaluación y Planificación del Servicio Canario de Salud (SESCS), Santa Cruz de Tenerife, 38109 Spain; 14 Institute for Applied Microelectronics (IUMA), Universidad de Las Palmas de Gran Canaria (ULPGC), Las Palmas de Gran Canaria, 35017 Spain; 15 Department of Psychiatry, Hospital Universitario Dr. Negrín, Las Palmas de Gran Canaria, 35019 Spain; 16 Universidad Fernando Pessoa Canarias, Las Palmas de Gran Canaria, Spain; 17 Clinical Analysis Department, Hospital Universitario Dr. Negrín, Las Palmas de Gran Canaria, 35019 Spain; 18 Asociación Española Contra el Cáncer (AECC), Delegación de Las Palmas, Spain

**Keywords:** Chemotherapy-induced peripheral neuropathy, Ozone therapy, Paraesthesia, Numbness, Tingling, Side effects, Quality of life, Anxiety and depression, Hyperspectral imaging, Oxidative stress

## Abstract

**Background:**

Chemotherapy-induced peripheral neuropathy (CIPN) is a frequent, disabling side effect of taxanes and platinum drugs, often compromising quality of life and treatment continuity. Existing therapies are few and largely ineffective. Given this unmet need, our prior experience suggests ozone therapy may offer clinical benefit as an adjuvant treatment.

**Methods:**

OzoParQT is a Phase II–III randomized, triple-blind trial including 42 adults (≥ 18 years) with any cancer and Grade ≥ 2 CIPN (numbness and/or tingling) lasting ≥ 3 months. Eligible patients must be off neurotoxic chemotherapy for ≥ 3 months, with stable/remitted disease and ≥ 6-month life expectancy. Participants will be randomized (1:1) to Ozone or Control (placebo) groups. All patients will continue standard care by their oncologists and undergo 40 rectal insufflation sessions over 16 weeks (3×/week for 8 weeks, then 2×/week). The Ozone group receives O₃/O₂ (10–30 µg/mL); the Control group receives O₂ (O_3_/O_2_: 0 µg/mL). Volumes range from 180 to 300 mL as tolerated.

The primary objectives are to evaluate the effect of adding ozone on change from baseline at week 28 (end of follow-up) in: i) patients' self-perceived level of paraesthesia (numbness and/or tingling), and ii) patients' self-perceived health-related quality of life (HRQoL).

Secondary objectives include evaluating the effect of ozone on: i) additional direct costs, ii) evolution of neuropathy symptoms (CTCAE v5.0, QLQ-CIPN20), iii) evolution of quality of life (EQ-5D-5L, and QLQ-C30), iv) evolution of anxiety and depression, v) evolution of biochemical parameters related to oxidative stress and chronic inflammation, vi) evolution of infrared images and spectral signatures (450 to 900 nm) in hyperspectral images obtained from hands and feet, and vii) toxicity of rectal ozone treatment. Except for direct costs and toxicity, all variables will be assessed at week 16 (end of insufflations) and week 28 (end of follow-up). Masking will be triple: participant, care provider, and outcomes assessor.

**Discussion:**

This study aims to provide robust evidence on the effectiveness and cost-effectiveness of ozone therapy as an adjuvant treatment for CIPN, a condition with very limited therapeutic options. The trial will clarify whether ozone therapy can significantly improve patient-reported symptoms and quality of life, potentially leading to a new management strategy with low morbidity.

**Trial registration:**

EU CT ID: 2024-517196-20-00. ClinicalTrials.gov Identifier NCT06706544, Registered January 22, 2025. https://clinicaltrials.gov/study/NCT06706544.

**Supplementary Information:**

The online version contains supplementary material available at 10.1186/s12885-025-15399-9.

## Background

Chemotherapy-induced peripheral neuropathy (CIPN) represents a significant challenge in oncology, affecting a large proportion of patients undergoing neurotoxic chemotherapy. Its prevalence varies depending on the chemotherapy regimen; however, it can be as high as 70%−100% in patients treated with taxanes or platinum-based regimens [1]. CIPN manifests with various symptoms, including alterations in proprioception, thermal sensitivity, pain, and loss of strength; however, paraesthesia, such as numbness and tingling, are almost universally present. Many times, the severity of CIPN can significantly diminish patients’ health-related quality of life (HRQoL) and require a reduction in chemotherapy dose or even its interruption, thereby compromising tumour control. Furthermore, CIPN can become a chronic condition, persisting for months or even years after the completion of chemotherapy, and may be associated with or exacerbate other symptoms like anxiety, depression, insomnia, and fatigue [2–4]. Currently, the prophylactic and therapeutic options for managing CIPN are notably limited and often possess a low degree of effectiveness. For instance, scientific evidence only supports the use of duloxetine for pain control in CIPN, and even then, it offers only a “modest magnitude of its benefit” (a 0.7-point improvement on a 10-point scale, which disappears one week after stopping treatment) with a “moderate degree” recommendation [3, 5]. While other treatments such as amitriptyline, gabapentin, and pregabalin are commonly used for paraesthesia due to CIPN, the American Society of Clinical Oncology (ASCO) Clinical Guideline has stated that there is insufficient evidence to recommend their use outside of randomized controlled trials (RCTs) [3, 6, 7]. This significant unmet medical need has led ASCO to establish the development and evaluation of new strategies to mitigate and manage the chronic side effects of cancer treatments, including CIPN, as an area of urgent research [8].

The pathophysiological mechanisms underlying CIPN, among many others, involve local processes such as ischemia, apoptosis induced by increased reactive oxygen species and oxidative stress, and an increase in pro-inflammatory mediators [6, 9–11].

Ozone (O_3_), a highly reactive compound, is produced from medical-grade oxygen (O_2_) using specialized generators. Medical ozone is typically applied as a gas mixture of O_3_/O_2_, where O_2_ constitutes the vast majority (95–99%). The mechanism of action of ozone treatment (O3T) is indirect, by a hormetic effect. O3T induces a rapid, “transient, controlled and moderate” local oxidative stress, which in turn triggers an adaptive response in the tissues and organism. This adaptive response involves the activation of nuclear transcription factors, such as Nrf2, leading to an increase in antioxidant enzymes and a decrease in pro-inflammatory mediators, thereby exerting an anti-inflammatory effect [12, 13]. Systemic administration of O_3_/O_2_ (particularly via autohemotherapy and rectal insufflation) has been shown to improve tissue oxygenation, decrease inflammation, and modulate oxidative stress, which are relevant to CIPN pathophysiology [14, 15]. Reviews of the mechanisms of action of O3T on CIPN have been previously published by our group [16, 17] and other authors [18].

Given the limited therapeutic options for CIPN, it is reasonable to explore other treatments based on their mechanisms of action or their utility in similar conditions. Our group’s preliminary studies with rectal O3T in patients with CIPN have shown promising results, including long-term reduction in pain [19], numbness and tingling [20] and other symptoms such as grade of toxicity and quality of life, anxiety and depression, as we have summarized in a recent review [17]. Rectal administration of O_3_/O_2_ has a long history, dating back to 1936, and is considered to have minimal risks when properly performed. The most common side effect reported is mild, transient meteorism or bowel bloating, which typically resolves spontaneously, as stated in our studies and also described by other authors [21, 22].

This randomized, triple-blind clinical trial is therefore designed to rigorously evaluate the effectiveness and cost-effectiveness of adding rectal O3T to the usual management of patients with CIPN, focusing on patient-centred outcomes such as self-perceived paraesthesia and HRQoL.

## Methods/design

### Aim

The primary aim of this study is to confirm the effectiveness of adding O3T to the usual management of patients with paraesthesia (numbness and/or tingling) secondary to CIPN on their self-perceived level of paraesthesia and HRQoL. Secondary aims include evaluating the impact of O3T on direct costs, sensory neuropathy evolution, anxiety and depression, biochemical markers of oxidative stress and inflammation, infrared images and hyperspectral signatures of hands and feet, and the toxicity of rectal O3T.

### Study design

This is a Phase II-III randomized, triple-blind, parallel-assignment clinical trial. The blinding will apply to the participant, the care provider (oncologists), and the outcomes assessor (researchers performing biochemical and functional tests, staff collecting economic data, and statisticians).

### Study setting

The study will be conducted at the Chronic Pain Unit of the Hospital Universitario de Gran Canaria Dr. Negrín (HUGCDN) in Las Palmas, Spain. Collaborating centres include Complejo Hospitalario Universitario Insular Materno Infantil (CHUIMI), Fundación Canaria Instituto de Investigación Sanitaria de Canarias (FIISC), Instituto Universitario de Microelectrónica Aplicada (IUMA) de la Universidad de Las Palmas de Gran Canaria (ULPGC), Grupo BioPharm de la ULPGC, Asociación Española Contra el Cáncer (AECC) in Las Palmas, and Servicio de Evaluación del Servicio Canario de Salud (SESCS) in Santa Cruz de Tenerife.

### Study population

Adult patients with a clinical diagnosis of persistent paraesthesia (numbness, tingling) secondary to CIPN, referred to the Chronic Pain Unit of the HUGCDN for evaluation of O3T, who fulfil all inclusion criteria, meet none of the exclusion criteria, and provide signed informed consent for participation in this RCT.

### Inclusion criteria

1. Adults aged 18 years or older.
2. Previous treatment with any chemotherapy for any tumour.
3. Clinical diagnosis of paraesthesia (numbness, tingling) secondary to CIPN, with toxicity Grade > = 2 (moderate symptoms and/or limitation in instrumental activities of daily living) according to the Common Toxicity Criteria for Adverse Events (CTCAE) from the National Cancer Institute of EEUU, v.5.0, for > = 3 months.
4. Without neurotoxic chemotherapy for > = 3 months.
5. Cancer disease is stable or in remission.
6. Life expectancy > = 6 months.
7. For women of childbearing potential, a negative serum or urine pregnancy test at screening and acceptance of appropriate contraceptive methods from 14 days prior to the first O3T session until 14 days after the last one.
8. Signed and dated study-specific informed consent.

### Exclusion criteria

1. Age < 18 years.
2. Lactating, pregnant, or suspected pregnant women, or women of childbearing potential not using adequate contraceptive methods.
3. Suspected symptoms due to diabetic or compressive neuropathy.
4. Severe psychiatric disorders.
5. Inability to complete quality of life questionnaires.
6. Creatinine elevation > 5 times the maximum limit of normal.
7. Hemodynamically or clinically unstable patients, or those requiring urgent or short-term interventional measures.
8. Neoplasia in progression requiring recent initiation of systemic treatment (or maintenance) with neurotoxic chemotherapy.
9. Life expectancy (for any reason) < 6 months.
10. Known allergy to ozone, known glucose 6 phosphate dehydrogenase (G6PD) deficiency, or hemochromatosis.
11. Contraindications or impossibility for rectal ozone treatment or to attend regularly to the treatment.
12. Not meeting any of the inclusion criteria.

### Interventions

All patients will receive the usual symptomatic treatment, management, and follow-up from their oncologists. Additionally, all patients will undergo a standardized rectal gas insufflation procedure.

1. Ozone Group: Patients will receive O_3_/O_2_ gas mixture by rectal insufflation. The O_3_/O_2_ concentration will start at 10 µg/mL and increase by 5 µg/mL every 2 sessions until it reaches 30 µg/mL by the 9th session, after which it will be maintained until week 16. The volume will start at 180 mL and will be progressively increased to 300 mL if tolerated. The total ozone dose will range from 1800 µg to 9000 µg.
2. Control Group (Placebo): Patients will receive O_2_ only (O_3_/O_2_ concentration: 0 µg/mL) using the same procedure, schedule, and volume as the Ozone group.

In both groups, the procedure will consist of 40 sessions administered over 16 weeks, with 3 sessions/week for the first 8 weeks and 2 sessions/week for the last 8 weeks. The procedure involves the placement of a rectal probe with lubricant, with patients advised to have an empty bladder and rectum. Treatment will be performed in the Chronic Pain Unit of HUGCDN.

Patients will be followed up for 12 weeks (3 months) after the end of O3T. The total duration of the study for each patient is 28 weeks. Planned duration for each patient’s participation will last 28 weeks (16 weeks of procedure with O_3_/O_2_ insufflations and 12 weeks of follow-up). The total duration of the project is planned for 60 months.

### Variables and outcomes

All variables (except direct hospital costs and toxicity) will be assessed at baseline (week 0), at the end of O_3_/O_2_ insufflation (week 16), and at the end of follow-up (week 28).

### Primary outcome measures

They will be self-evaluated by the patients.

1. Change from baseline in “numbness and tingling” self-perceived by patients at the end of follow-up: Self-reported evaluation of the percentage of “numbness and/or tingling” regarding the basal level, from 100% (basal level, 0% improvement) to 0% (no numbness and tingling, 100% improvement), at the end of follow-up (week 28). The percentage of improvement in any kind of symptoms (including numbness and tingling) is a usual clinical record in our Chronic Pain Unit, and it is a patient-reported outcome (PRO) easily self-evaluated by patients.
2. Change from baseline in HRQoL using the EQ-5D-5 L questionnaire (developed by the EuroQol Group) self-perceived by patients at the end of follow-up: Self-reported evaluation of: (a) 5 physical and emotional items scored in five levels, from 1 (Best: I have no problem) to 5 (worst: I have an extreme problem or I am unable to…) and (b) additional self-assessment of health by a visual analogue scale (VAS) (0 = worst health patient can imagine, 100 = best health patient can imagine), at the end of follow-up (week 28).

### Secondary outcome measures

1. Direct hospital costs: The direct expenses incurred by the hospital in providing services during the 28 weeks for the study (in euros).
2. Change from baseline in “numbness and tingling” self-perceived by patients at the end of O3T (week 16).
3. Change from baseline in HRQoL using the EQ-5D-5 L questionnaire, self-perceived by patients at the end of O3T (week 16).
4. Changes from baseline in the Grade of toxicity of paraesthesia (numbness, tingling) according to the CTCAE v.5.0. scale (from the National Cancer Institute of EEUU). Range from Grade 0 (asymptomatic or mild symptoms) to Grade 3 (severe symptoms, limiting self-care activities in daily life). Assessed at the end of O3T (week 16) and at the end of follow-up (week 28).
5. Changes from baseline in the Grade of toxicity of sensory neuropathy according to the CTCAE v.5.0. scale. Range from Grade 0 (asymptomatic or mild symptoms) to Grade 4 (life-threatening consequences, urgent intervention indicated). Assessed at the end of O3T (week 16) and at the end of follow-up (week 28).
6. Changes from baseline in the degree of neuropathy according to the QLQ-CIPN20 scale from the European Organization for Research & Treatment in Cancer (EORTC). It is evaluated through 20 items grouped into 3 dimensions: sensitive, motor, and autonomic. Range: each item is scored from 1 (nothing) to 4 (a lot). The total score for each dimension is transformed into a score from 0 to 100, with 0 being the best state and 100 the worst. Assessed at the end of O3T (week 16) and at the end of follow-up (week 28).
7. Changes from baseline in the HRQoL according to the QLQ-C30 questionnaire from the EORTC: Self-reported evaluation of 30 items that measure several scales and symptoms. Range (after standardization): from 0 (worst for overall health and function, best for symptoms) to 100 (best for overall health and functions, worst for symptoms). Assessed at the end of O3T (week 16) and at the end of follow-up (week 28).
8. Changes from baseline in levels of anxiety and depression according to the Hospital Anxiety and Depression Scale (HADS). HADS is a self-administered questionnaire that assesses 14 items/symptoms of anxiety (7) and depression (7) experienced by patients. Each item is scored from 0 (better, no alteration) to 3 (worse level of alteration). For each symptom (anxiety or depression), the overall score is from 0 (better, no anxiety or depression) to 21 (worse, very severe anxiety or depression). Assessed at the end of O3T (week 16) and at the end of follow-up (week 28).
9. Changes from baseline in biochemical parameters of oxidative stress (superoxide dismutase, glutathione, glutathione peroxidase, and free radicals). Assessed at the end of O3T (week 16) and at the end of follow-up (week 28).
10. Changes from baseline in biochemical parameters of inflammation cytokines. Assessed at the end of O3T (week 16) and at the end of follow-up (week 28).
11. Changes from baseline in spectral signatures (450 to 900 nm) of hyperspectral images and temperature variations in infrared images obtained from hands and feet. Assessment of the percentage of reflectance for each wavelength of the hyperspectral and thermal images obtained with specific devices. Assessed at the end of O3T (week 16) and at the end of follow-up (week 28).
12. Toxicity of rectal O3T, recorded according to CTCAE v5.0.

A schematic diagram illustrating the time schedule of enrolment, interventions, assessments, and participant visits is included as Table 1.

**Table 1:** Time schedule of enrolment, interventions, assessments, and visits

Week	0	1–8	9–16	17–28 (Follow-up)
Enrolment	Screening, Baseline assessments			
Interventions		Rectal insufflation: 3 sessions/week (total 24 sessions)	Rectal insufflation: 2 sessions/week (total 16 sessions)	No experimental intervention (follow-up only)
		Ozone Group: O_3_/O_2_ gas, increasing concentration (10→30 µg/mL)	Ozone Group: O_3_/O_2_ gas at 30 µg/mL	
		Control Group: O_2_ only (O_3_/O_2_: 0 µg/mL)	Control Group: O_2_ only (O_3_/O_2_: 0 µg/mL)	
Procedure Details		Rectal probe insertion, volume 180–300 mL if tolerated	Same as weeks 1–8	
Assessments	Baseline: Primary and Secondary outcomes: - umbness/tingling - HRQoL EQ-5D-5 L and QLQ-C30 - Toxicity grades (CTCAE v5.0) - Neuropathy QLQ-CIPN20 - HADS - Biochemical parameters (oxidative stress, cytokines) - Hyperspectral and infrared imaging		End of O3T: Re-assessment of Primary and Secondary outcomes: - Same as basal (week 0)	End of follow-up Re-assessment of Primary and Secondary outcomes: - Same as basal (week 0). Additionally: - Hospital costs - Rectal O3T toxicity
Follow-up Visits				Regular follow-up visits for 12 weeks post-treatment

Key assessments at Week 0 (Baseline), Week 16 (end of intervention), and Week 28 (end of follow-up). Secondary variables include hospital costs, toxicity, quality of life, biochemical parameters, neuropathy signs, mental health, and imaging analyses. Total patient duration: 28 weeks. Total project duration: 60 months

*CTCAE* Common Terminology Criteria for Adverse Events, *EORTC* European Organization for Research and Treatment of Cancer, *HADS* Hospital Anxiety and Depression Scale, *HRQoL* Health-Related Quality of Life, *O3T* Ozone Treatment, *QLQ-C30* Quality of Life Questionnaire-Core 30, *QLQ-CIPN20* Quality of Life Questionnaire-Chemotherapy-Induced Peripheral Neuropathy 20-item scale, *VAS* Visual Analog Scale

### Randomization and masking

A stratified randomization will be carried out based on sex to ensure a homogeneous distribution between the two arms. Randomization will be managed by a statistician, who will be independent of those who analyse the results. Opaque, sealed envelopes containing the assigned treatment group (coded as Group A or Group B) will be used. The principal investigator will maintain a record of patient names, randomization dates, and code numbers. However, the key linking the code to the actual treatment (O_3_ or O_2_) will be known only to the randomization investigators and Chronic Pain Unit staff, remaining hidden until the analysis is completed. This ensures triple blinding of participants, care providers (oncologists), and outcomes assessors. The treatment received by each patient will not be known by any of the following: (i) the patient, (ii) the oncologists who treat and follow the patient on a regular basis, (iii) researchers who carry out biochemical determinations or functional tests, (iv) the staff (from the Hospital’s Accounting Department) who obtain the economic data, (v) the researchers of statistical analysis.

### Sample size calculation

The sample size of 42 patients (21 per group) was calculated using GRANMO software (https://www.datarus.eu/aplicaciones/granmo/). This is based on an expected clinically relevant reduction in symptoms (>50% decrease in numbness and tingling from baseline) in at least 50% of the ozone group patients, compared to less than 10% in the placebo group, with 80% power, a one-sided alpha of 0.05, a 1:1 ratio, and accounting for 10% possible loss to follow-up. Data based on our preliminary results [20].

#### Strategies for achieving adequate participant enrolment to reach the target sample size in the clinical trial

Engaging referring clinicians: Collaborate with oncologists, general practitioners, or other relevant specialists to identify and refer eligible patients. Patient outreach and education: Use flyers, informational sessions, social media, or community events to raise awareness about the study and its potential benefits. Providing clear informed consent materials: Ensure patients fully understand the study, its procedures, and potential benefits/risks, to increase willingness to participate. Collaboration with patient associations, such as the Las Palmas delegation of the Spanish Association Against Cancer (AECC). Monitoring recruitment progress regularly: Track enrolment metrics every 2 months to identify bottlenecks early and adjust strategies as needed.

#### Withdrawal of individual subjects

Subjects can leave the study at any time for any reason, if they wish to do so. Then, they will continue with the usual symptomatic treatment from their oncologists, as they did before participating in the trial. Their decision will not otherwise have any consequences for their further therapy.

#### Replacement of individual subjects after withdrawal

Patients will not be replaced after withdrawal after randomization.

#### Premature termination of the study

The study can be prematurely terminated after the interim safety analysis, conducted when patient #21 completes follow-up, if unforeseen serious complications or differences are statistically and clinically relevant in the main clinical outcomes, as evaluated by the principal investigator and the Ethics Committee.

Allocated interventions may be discontinued or modified for a participant under the following criteria:

- In response to harms: If a participant experiences adverse events or toxicity deemed related to the intervention that are severe, unexpected, or pose a safety risk, the intervention will be paused, reduced, or stopped as clinically appropriate.
- Participant request: Participants have the right to withdraw from or discontinue the intervention at any time without penalty or loss of benefits.
- Improving or worsening disease: If a participant’s clinical condition changes significantly—either substantial improvement making continued treatment unnecessary, or worsening disease requiring alternative therapies—the intervention may be modified or discontinued based on clinical judgment and protocol guidelines.

All such decisions will be documented, and participants will continue to be followed for outcome assessment.

#### Plans for assessment and collection of outcomes

Baseline, and other trial data: outcome, baseline, and other trial data will be collected using standardized case report forms (CRFs) developed specifically for this study. Data collection will be performed by trained personnel following detailed standard operating procedures (SOPs) to ensure consistency and accuracy. Assessors will undergo specific training sessions to minimize inter-observer variability and promote data quality. When applicable, duplicate measurements will be conducted to verify reliability.

Patient-reported outcomes, including symptom severity and quality of life, will be assessed using validated instruments such as the VAS, the CTCAE grade of toxicity, or the EORTC QLQ-CIPN20 questionnaires, which have demonstrated good reliability and validity in oncology populations.

Laboratory tests and clinical assessments will follow standard hospital protocols and performed in certified laboratories.

#### Plans to promote participant retention and complete follow-up

To promote participant retention and ensure complete follow-up, the study will implement several strategies, including regular contact with participants through phone calls, reminder messages, and flexible scheduling of visits to accommodate individual needs. Participants will be provided with clear information about the importance of follow-up regardless of adherence to the intervention protocol.

For participants who discontinue the intervention or deviate from the protocol, efforts will be made to collect key outcome data, including primary and secondary endpoints such as symptom severity (e.g., VAS scores), quality of life measures (e.g., EQ-5D-5 L, or QLQ-CIPN20), and any reported adverse events. These participants will continue to be included in the intention-to-treat analysis to preserve the integrity of the study findings.

#### Plans for data entry, coding, security, and storage

Data will be entered into a secure, password-protected electronic database by trained personnel. The database with the patients’ clinical information and case report forms (CRFs) will be located on the “Hospital Universitario de Gran Canaria Dr. Negrín” intranet. To ensure data quality, the study will implement double data entry for critical variables and perform regular range and consistency checks to identify and correct discrepancies. All data will be coded using standardized coding systems, and personal identifiers will be removed or anonymized to protect participant confidentiality.

Access to the database will be restricted to authorized study staff only. Regular backups will be conducted, and data will be stored on encrypted servers in compliance with institutional and regulatory requirements. The responsibilities for data security and confidentiality are assumed by the Data Security Section of the Information and Communications Service of the General Secretariat of the Canary Islands Health Service.

If not fully detailed in the study protocol, additional information on data management procedures will be available in the study’s Data Management Plan (DMP), which can be provided upon request.

### Statistical analysis

A double analysis will be performed: “by intention to treat” (all included patients) and “by protocol” (only patients who completed the study in their assigned group). Normality will be assessed using the Kolmogorov-Smirnov test. Depending on data distribution, parametric or non-parametric tests will be used for comparisons between groups. Contingency tables will use Fisher’s exact test. Variables with more than two follow-up assessments will be analysed using a mixed generalized linear model with repeated measures. Bonferroni adjustment will be applied for multiple comparisons, with a p-value < 0.05 considered significant.

An interim safety analysis will be conducted when patient #21 completes follow-up, with the possibility of premature study termination if unforeseen serious complications or differences are statistically and clinically relevant in the main clinical outcomes after evaluation by the Ethics Committee.

For participants who deviate from the protocol or discontinue the intervention, all available outcome data will be included in the analysis.

#### Composition of data monitoring committee (DMC)

A DMC is not deemed necessary for this study due to its relatively low-risk nature, the non-invasive intervention (rectal ozone therapy), and the short to moderate duration of participant involvement. The study does not involve high-risk treatments or vulnerable populations, and the expected adverse events are minimal and well-characterized based on prior clinical experience.

Safety will be monitored internally by the principal investigator and the clinical research team, who will review adverse events and protocol adherence on an ongoing basis. In the event of any serious or unexpected adverse events, they will be reported immediately to the ethics committee and relevant regulatory authorities, in accordance with standard procedures.

Given these factors, the establishment of an independent DMC is not required. Full details of the safety monitoring procedures are included in the study protocol.

However, safety and participant well-being will be continuously monitored by the principal investigator and the clinical research team. In the event of unexpected adverse events or safety concerns, the research team will review the data and, if necessary, consult the Ethics Committee to determine whether the trial should be modified or terminated. Access to any such safety data will be restricted to the research team responsible for monitoring adverse events.

All adverse events (AEs), whether solicited or spontaneously reported, will be systematically collected throughout the study period. Participants will be asked about any symptoms or changes in health during each scheduled visit, and any reported AEs will be documented in the CRFs.

Each AE will be assessed by the clinical research team in terms of severity, duration, and relationship to the study intervention. Serious adverse events (SAEs) will be reported immediately to the ethics committee and relevant regulatory authorities, in accordance with applicable guidelines.

All AEs and SAEs will be managed according to standard clinical practice. Appropriate medical care will be provided as needed, and participants will be followed until the resolution or stabilization of the event. The principal investigator will be responsible for ensuring that all safety information is reviewed regularly and that appropriate action is taken if safety concerns arise.

No formal auditing is planned for this trial due to its low-risk nature and limited scale. However, internal monitoring procedures will be implemented to ensure compliance with the protocol, GCP guidelines, and regulatory requirements. Monitoring activities will be carried out by designated study personnel who are not involved in patient recruitment or intervention delivery, to maintain a degree of independence. These procedures will include periodic review of consent forms, data entry accuracy, and adverse event reporting.

If requested by regulatory authorities or the ethics committee, additional independent audits may be conducted. Any such audits would be carried out by individuals or organizations independent from the investigators and the sponsor.

### Ethical concern

The study protocol has already received approval from the appropriate Research Ethics Committee/Institutional Review Board (REC/IRB). Full details of the ethical approval, including the name of the committee, approval number, and date of approval, are provided in the Declarations section of the manuscript. Any protocol amendments will be submitted for further ethical review as required.

Any important protocol modifications—such as changes to eligibility criteria, outcomes, or statistical analyses—will be promptly communicated to all relevant parties. These include the investigators, the REC/IRB, trial participants (when applicable), the trial registry, and, if necessary, regulatory authorities and journals.

All amendments will be documented clearly, and updates will be made in the trial registry entry. Investigators will be informed through official written communication, and participants will be re-consented if the changes affect their participation or safety.

Informed consent will be obtained by trained members of the clinical research team, specifically designated by the principal investigator. The process will take place in a private setting, ensuring adequate time and opportunity for potential participants to ask questions and fully understand the study objectives, procedures, risks, and benefits.

Consent will be obtained in writing before any study-related procedures are initiated. If a participant is unable to provide consent directly, an authorised legal representative or surrogate may do so, in accordance with local regulations and ethical guidelines. All consent procedures will follow GCP standards and be documented appropriately.

#### Confidentiality

Personal information about potential and enrolled participants will be collected using secure, standardized case report forms and stored in a password-protected electronic database with restricted access. Each participant will be assigned a unique study identification code to anonymize data; identifiable information will be stored separately from clinical data.

Data sharing will be limited to authorized study personnel and, when required, ethics committees or regulatory authorities, always in compliance with data protection regulations. During the trial, all data handling will follow GCP and institutional policies to ensure confidentiality. After the trial, data will be securely archived for the period required by law and institutional guidelines and then disposed of or anonymized appropriately.

#### Dissemination policy

The trial results will be communicated to participants, healthcare professionals, and the public through multiple channels. A summary of the study findings will be provided to participants in a clear and accessible format after the trial’s completion. Results will also be submitted for publication in peer-reviewed scientific journals and presented at relevant conferences.

Additionally, the trial outcomes will be reported in recognized clinical trial registries in accordance with regulatory requirements. Data sharing will comply with ethical standards and participant confidentiality.

There are no publication restrictions imposed by the sponsor; investigators retain full rights to publish and disseminate the study results.

## Discussion

This study addresses a critical unmet need in oncology: the effective management of CIPN [2]. Current treatment options are limited and often offer only modest benefits [1]. Thereafter, robust RCTs investigating novel strategies like O3T are essential. The OzoParQT trial was designated to provide high-level evidence regarding the efficacy and safety of rectal O3T, potentially establishing it as a new, low-morbidity treatment option for patients suffering from chronic numbness and tingling due to CIPN.

The preliminary results from our research group, which demonstrate long-term improvements in pain, quality of life, anxiety, depression, and reduction in numbness/tingling with rectal O3T, provide a strong rationale for this comprehensive Phase II-III trial [19, 20]. These findings, coupled with the established mechanistic understanding of ozone’s antioxidant and anti-inflammatory effects that directly target CIPN pathophysiology, suggest a high potential for clinical benefit [17].

A key strength of this study is its triple-blinded, randomized design, which minimizes bias and enhances the reliability of the results. The inclusion of patient-reported outcomes for paraesthesia and HRQoL as primary endpoints aligns with current recommendations to prioritize patients’ own assessment of their symptoms, which often differs from physician assessment.

The comprehensive evaluation of secondary outcomes, including cost-effectiveness, biochemical markers, and hyperspectral signatures, will provide a holistic understanding of ozone’s impact and its underlying biological mechanisms. The use of hyperspectral imaging is an innovative aspect, as it has shown potential in assessing functional alterations such as those causing CIPN symptoms and has been used by our group to demonstrate perfusion changes [23].

From a practical perspective, rectal ozone insufflation is an inexpensive and relatively low-risk procedure, with reported side effects being typically mild and transient, such as meteorism [22]. This contrasts favourably with the potential side effects and limited efficacy of existing pharmacological interventions for CIPN. The adaptive dosing of ozone concentrations is designed to optimize the therapeutic effect while maintaining safety. Furthermore, the protocol ensures that all patients will continue to receive their usual oncological and symptomatic care, minimizing any ethical concerns related to withholding standard treatments. The interim safety analysis will serve as an additional safeguard for patient well-being.

While preliminary data are encouraging [19, 20], the definitive value of O3T for CIPN can only be confirmed through a rigorous randomized trial like OzoParQT. The results of this study are expected to inform clinical guidelines and potentially integrate O3T into the standard management of CIPN, improving the HRQoL for a significant population of cancer survivors, currently without successful treatment.

## Supplementary Information

Supplementary Material 1.

## Data Availability

No datasets were generated or analysed during the current study.

## Abbreviations

AE: Adverse Events
AECC: Asociación Española Contra el Cáncer
AEMPS: Spanish Medicines Agency
ASCO: American Society of Clinical Oncology
CRF: Case Report Forms
CIPN: Chemotherapy-induced peripheral neuropathy
CTCAE: Common Toxicity Criteria for Adverse Events from the National Cancer Institute of EEUU
DMC: Data Monitoring Committee
DMP: Data Management Plan
EMA: European Medicines Agency
EORTC: European Organization for Research & Treatment in Cancer
G6PD: Glucose 6 phosphate dehydrogenase
GCP: Good Clinical Practice
HADS: Hospital Anxiety and Depression Scale
HRQoL: Health-related quality of life
HUGCDN: Hospital Universitario de Gran Canaria Dr. Negrín
IPD: Individual participant data
O_2_: Oxygen
O_3_: Ozone
O3T: Ozone treatment
REC/IRB: Research Ethics Committee/Institutional Review Board
RCT: Randomized controlled trial
REEC: Spanish Clinical Studies Registry
SAE: Serious adverse events
SOP: Standard Operating Procedures
VAS: Visual analogue scale
