# Supplementary material for: Effectiveness and cost-effectiveness of ozone treatment in patients with paraesthesia (numbness, tingling) secondary to chemotherapy-induced peripheral neuropathy: randomized, triple-blind clinical trial (OzoParQT)
Source: BMC Cancer. 2026 Jan 22;26:260. doi: 10.1186/s12885-025-15399-9 (PMC12911077; doi:10.1186/s12885-025-15399-9)
Supplement: Supplementary file 1 — Supplementary Material 1. [file 12885_2025_15399_MOESM1_ESM.doc]

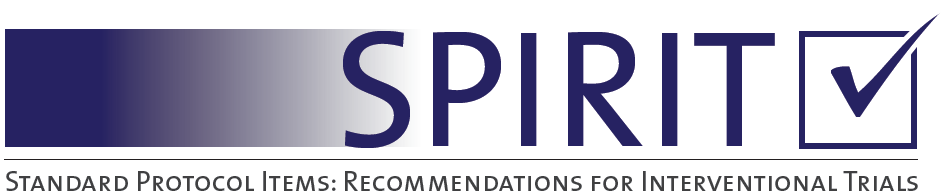


SPIRIT 2013 Checklist: Recommended items to address in a clinical trial protocol and related documents*

| Section/item | ItemNo | Description |
| --- | --- | --- |
| **Administrative information** | | |
| Title | 1 | Effectiveness and Cost-effectiveness of Ozone Treatment in Patients with Paraesthesia (Numbness, Tingling) Secondary to Chemotherapy-induced Peripheral Neuropathy: Randomized, Triple-blind Clinical Trial (OzoParQT) |
| Trial registration | 2a | EU CT ID: 2024-517196-20-00. ClinicalTrials.gov Identifier: NCT06706544, Registered: January 22, 2025. *https://clinicaltrials.gov/study/NCT06706544* |
|  | 2b | CTIS2024-517196-20-00 |
| Protocol version | 3 | Last Update Submitted that met QC Criteria 2025-02-07 |
| Funding | 4 | This study is supported (after peer review) by a national grant PI 23/01324 from the Instituto de Salud Carlos III (Spanish Ministry of Science and Innovation, Madrid, Spain) and European Regional Development Fund—ERDF), and a regional grant PIFIISC24/37 from the Fundación Canaria Instituto Investigación Sanitaria de Canarias (FIISC), Las Palmas, Spain. It is also supported by a dedicated grant CIGC’23-24 from the Cabildo de Gran Canaria, Las Palmas, Spain. |
| Roles and responsibilities | 5a | Bernardino Clavo ^1,2,3,4,5,6,7,8,*^, Angeles Cánovas-Molina^1,2,4^, Sara Cazorla-Rivero^1,4^, Gregorio Martínez-Sánchez^9^, Saray Galván^10^, Gretel Benítez^11^, Mario Federico^1,3,4^, Himar Fabelo^1,4,14^, Jesús M González-Martín^1,4^,, M.A. García-Bello^4,12,13^, Elba Lago-Moreno^1,2,4^, Carla Antonilli^10^, Avi Ramchandani^11^, Juan A Díaz-Garrido^4,15,16^, Minerva Navarro^2,4^, Alexia Suárez-Cabrera^17^, Ruth Martín-Alfaro^18^, Haidé Hernández-López^19^, Gustavo Marrero-Callico^14^, Francisco Rodríguez-Esparragón^1.4.6.7,*^  ^1^ Research Unit, Hospital Universitario de Gran Canaria Dr. Negrín, 35019 Las Palmas de Gran Canaria, Spain.  ^2^ Chronic Pain Unit, Dr. Negrín University Hospital, 35019 Las Palmas de Gran Canaria, Spain.  ^3^ Radiation Oncology Department, Hospital Universitario Dr. Negrín, 35019 Las Palmas de Gran Canaria, Spain.  ^4^ Fundación Canaria Instituto de Investigación Sanitaria de Canarias (FIISC), Las Palmas de Gran Canaria/Tenerife, Spain.  ^5^ University Institute for Research in Biomedicine and Health (iUIBS), Molecular and Translational Pharmacology Group, University of Las Palmas de Gran Canaria, 35016 Las Palmas de Gran Canaria, Spain;  ^6^ Instituto Universitario de Enfermedades Tropicales y Salud Pública de Canarias de la Universidad de La Laguna, 38296 La Laguna, Tenerife, Spain.  ^7^ CIBER de Enfermedades Infecciosas, Instituto de Salud Carlos III, 28029 Madrid, Spain  ^8^ Spanish Group of Clinical Research in Radiation Oncology (GICOR), 28290 Madrid, Spain  ^9^ Scientific Advisor, Freelance, 60126 Ancona, Italy  ^10^ Medical Oncology Department, Hospital Universitario de Gran Canaria Dr. Negrín, 35019 Las Palmas de Gran Canaria, Spain  ^11^ Medical Oncology Department, Complejo Hospitalario Universitario Insular Materno-Infantil de Gran Canaria, 35016 Las Palmas de Gran Canaria, Spain  ^12^ Network for Research on Chronicity, Primary Care, and Health Promotion (RICAPPS), Barcelona, Spain  ^13^ Servicio de Evaluación y Planificación del Servicio Canario de Salud (SESCS), 38109 Santa Cruz de Tenerife, Spain  ^14^ Institute for Applied Microelectronics (IUMA), University of Las Palmas de Gran Canaria (ULPGC), 35017 Las Palmas de Gran Canaria, Spain;  ^15^ Department of Psychiatry, Hospital Universitario Dr. Negrín, 35019 Las Palmas de Gran Canaria, Spain.  ^16^ Universidad Fernando Pessoa Canarias, Las Palmas de Gran Canaria, Spain.  ^17^ Hematology, Hospital Universitario Dr. Negrín, 35019 Las Palmas de Gran Canaria, Spain.  ^18^ Clinical Analysis Department, Hospital Universitario Dr. Negrín, 35019 Las Palmas de Gran Canaria, Spain.  ^19^ Asociación Española Contra el Cáncer (AECC), Delegación de Las Palmas, Spain  **Authors' Contributions**  BC serves as the Sponsor-Investigator, Study Chair, and Principal Investigator. FR-E contributed to the study design and serves as Study Director and Principal Investigator.  BC, AC-M, EL-M, and MN are responsible for ozone treatment. GM-S collaborates as an external O3T advisor in various regulatory, administrative, and manuscript writing processes. SG, GB, MF, CA, AR, and AS-C are oncologists and haematologists responsible for the conventional management of patients.  FR-E, SC-R, and RM-A will analyse the biochemical parameters. JMG-M and MAG-B are statisticians responsible for randomizing patients and analysing the results. JAD-G and HH-L are psychologists responsible for anxiety and depression analysis. HF and GM-C are contributing to the development and analysis of hyperspectral imaging aspects. All authors contributed to the drafting and critical revision of the manuscript and approved the final version. |
|  | 5b | **Name:** Bernardino Clavo, MD, PhD  **Phone Number:** 34928449278  **Email:** [bernardinoclavo@gmail.com](mailto:bernardinoclavo@gmail.com?subject=NCT06706544, OzoParQT, Ozone Treatment in Paresthesia (Numbness, Tingling) Secondary to Chemotherapy-induced Peripheral Neuropathy)  **Name:** Francisco Rodríguez-Esparragón, BSc, PhD  **Phone Number:** 34928449288  **Email:** [afrodesp@gmail.com](mailto:afrodesp@gmail.com?subject=NCT06706544, OzoParQT, Ozone Treatment in Paresthesia (Numbness, Tingling) Secondary to Chemotherapy-induced Peripheral Neuropathy) |
|  | 5c | Role of funders / Sponsors: only financial support  Sponsors cited in 5 b correspond to Scientific sponsors, that are full involved in: study design; collection, management, analysis, and interpretation of data; writing of the report; and the decision to submit the report for publication. |
|  | 5d | Coordinating centres:  Research Unit, Hospital Universitario de Gran Canaria Dr. Negrín, 35019 Las Palmas de Gran Canaria, Spain.  Chronic Pain Unit, Dr. Negrín University Hospital, 35019 Las Palmas de Gran Canaria, Spain.  Radiation Oncology Department, Hospital Universitario Dr. Negrín, 35019 Las Palmas de Gran Canaria, Spain.  Fundación Canaria Instituto de Investigación Sanitaria de Canarias (FIISC), Las Palmas de Gran Canaria/Tenerife, Spain.  University Institute for Research in Biomedicine and Health (iUIBS), Molecular and Translational Pharmacology Group, University of Las Palmas de Gran Canaria, 35016 Las Palmas de Gran Canaria, Spain;  Instituto Universitario de Enfermedades Tropicales y Salud Pública de Canarias de la Universidad de La Laguna, 38296 La Laguna, Tenerife, Spain.  Instituto de Salud Carlos III, 28029 Madrid, Spain  Spanish Group of Clinical Research in Radiation Oncology (GICOR), 28290 Madrid, Spain  Network for Research on Chronicity, Primary Care, and Health Promotion (RICAPPS), 08007 Barcelona, Spain  Servicio de Evaluación y Planificación del Servicio Canario de Salud (SESCS), 38109 Santa Cruz de Tenerife, Spain  Institute for Applied Microelectronics (IUMA), University of Las Palmas de Gran Canaria (ULPGC), 35017 Las Palmas de Gran Canaria, Spain;  Department of Psychiatry, Hospital Universitario Dr. Negrín, 35019 Las Palmas de Gran Canaria, Spain.  Universidad Fernando Pessoa Canarias, Las Palmas de Gran Canaria, Spain.  Asociación Española Contra el Cáncer (AECC), Delegación de Las Palmas, Spain |
| Introduction |  |  |
| Background and rationale | 6a | Description of research question:  Ozone therapy may offer clinical benefit as an adjuvant treatment of Chemotherapy-induced peripheral neuropathy  Participants will be randomized (1:1) to Ozone or Control (placebo) groups. All will continue standard care and undergo 40 rectal insufflation sessions over 16 weeks (3×/week for 8 weeks, then 2×/week). The Ozone group receives O₃/O₂ (10–30 µg/mL); the Control group receives O₂ (0 µg/mL). Volumes range from 180 to 300 mL as tolerated. |
|  | 6b | Explanation for choice of comparators: Oxygen is the vehicle of ozone |
| Objectives | 7 | The **primary objectives** are to evaluate the effect of adding ozone on change from baseline at week 28 (end of follow-up) in: i) patients' self-perceived level of paraesthesia (numbness and/or tingling), and ii) patients' self-perceived health-related quality of life (HRQoL).  **Secondary objectives** include evaluating the effect of ozone on: i) additional direct costs, ii) evolution of neuropathy symptoms (CTCAE v5.0, QLQ-CIPN20), iii) evolution of quality of life (EQ-5D-5L, and QLQ-C30), iv) evolution of anxiety and depression, v) evolution of biochemical parameters related to oxidative stress and chronic inflammation, vi) evolution of infrared images and spectral signatures in hyperspectral images obtained from hands and feet, and vii) toxicity of rectal ozone treatment. Except for direct costs and toxicity, all variables will be assessed at week 16 (end of insufflations) and week 28 (end of follow-up). Masking will be triple: participant, care provider, and outcomes assessor. |
| Trial design | 8 | OzoParQT is a Phase II–III randomized, triple-blind trial including 42 adults (≥18 years) with any cancer and Grade ≥2 CIPN lasting ≥3 months. Eligible patients must be off neurotoxic chemotherapy for ≥3 months, with stable/remitted disease and ≥6-month life expectancy. |
| Methods: Participants, interventions, and outcomes | | |
| Study setting | 9 | Academic hospital.  Hospital Universitario de Gran Canaria Dr. Negrín, 35019 Las Palmas de Gran Canaria, Spain. |
| Eligibility criteria | 10 | **Inclusion Criteria**   1. Adults aged 18 years or older. 2. Previous treatment with any chemotherapy for any tumour. 3. Clinical diagnosis of paraesthesia (numbness, tingling) secondary to CIPN, with toxicity Grade >= 2 (moderate symptoms and/or limitation in instrumental activities of daily living) according to the Common Toxicity Criteria for Adverse Events (CTCAE) from the National Cancer Institute of EEUU, v.5.0, for >= 3 months. 4. Without neurotoxic chemotherapy for >= 3 months. 5. Cancer disease is stable or in remission. 6. Life expectancy >= 6 months. 7. For women of childbearing potential, a negative serum or urine pregnancy test at screening and acceptance of appropriate contraceptive methods from 14 days prior to the first O3T session until 14 days after the last one. 8. Signed and dated study-specific informed consent.   **Exclusion Criteria**   1. Age < 18 years. 2. Lactating, pregnant, or suspected pregnant women, or women of childbearing potential not using adequate contraceptive methods. 3. Suspected symptoms due to diabetic or compressive neuropathy. 4. Severe psychiatric disorders. 5. Inability to complete quality of life questionnaires. 6. Creatinine elevation > 5 times the maximum limit of normal. 7. Hemodynamically or clinically unstable patients, or those requiring urgent or short-term interventional measures. 8. Neoplasia in progression requiring recent initiation of systemic treatment (or maintenance) with neurotoxic chemotherapy. 9. Life expectancy (for any reason) < 6 months. 10. Known allergy to ozone, known glucose 6 phosphate dehydrogenase (G6PD) deficiency, or hemochromatosis. 11. Contraindications or impossibility for rectal ozone treatment or to attend regularly to the treatment. 12. Not meeting any of the inclusion criteria. |
| Interventions | 11a | **Interventions**  All patients will receive the usual symptomatic treatment, management, and follow-up from their oncologists/haematologists. Additionally, all patients will undergo a standardized rectal gas insufflation procedure.   1. Ozone Group: Patients will receive O_3_/O_2_ gas mixture by rectal insufflation. The O_3_/O_2_ concentration will start at 10 µg/mL and increase by 5 µg/mL every 2 sessions until it reaches 30 µg/mL by the 9th session, and then be maintained until week 16. The volume will start at 180 mL and will be progressively increased to 300 mL if tolerated. The total ozone dose will range from 1800 µg to 9000 µg. 2. Control Group (Placebo): Patients will receive O_2_ only (O_3_/O_2_ concentration: 0 µg/mL) using the same procedure, schedule, and volume as the Ozone group.   In both groups, the procedure will consist of 40 sessions administered over 16 weeks, with 3 sessions/week for the first 8 weeks and 2 sessions/week for the last 8 weeks. The procedure involves the placement of a rectal probe with lubricant, with patients advised to have an empty bladder and rectum. Treatment will be performed in the Chronic Pain Unit of HUGCDN.  Patients will be followed up for 12 weeks (3 months) after the end of O3T. Total duration of the study: 289 weeks. Planned duration for each patient's participation will last 28 weeks (16 weeks of procedure with O_3_/O_2_ insufflations and 12 weeks of follow-up). The total duration of the project is planned for 60 months. |
|  | 11b | Criteria for discontinuing or modifying allocated interventions for a given trial participant: in response to harms, participant request, or improving/worsening disease.  Allocated interventions may be discontinued or modified for a participant under the following criteria:   - **In response to harms:** If a participant experiences adverse events or toxicity deemed related to the intervention that are severe, unexpected, or pose a safety risk, the intervention will be paused, reduced, or stopped as clinically appropriate. - **Participant request:** Participants have the right to withdraw from or discontinue the intervention at any time without penalty or loss of benefits. - **Improving or worsening disease:** If a participant’s clinical condition changes significantly—either substantial improvement making continued treatment unnecessary, or worsening disease requiring alternative therapies—the intervention may be modified or discontinued based on clinical judgment and protocol guidelines.   All such decisions will be documented, and participants will continue to be followed for outcome assessment. |
|  | 11c | Strategies to improve adherence to intervention protocols, and any procedures for monitoring adherence: Records of each intervention:  To improve adherence to intervention protocols, participants will receive thorough instructions and support from the study team at each visit. Regular scheduling and reminders for treatment sessions will be implemented to encourage consistent participation.  Adherence will be monitored through detailed records of each intervention session, including date, duration, and any deviations or issues encountered. These records will be maintained in case report forms and reviewed regularly by the research team to identify and address any adherence challenges promptly. |
|  | 11d | Relevant concomitant care and interventions that are permitted or prohibited during the trial: not applicable |
| Outcomes | 12 | **Variables and Outcomes**  All variables (except direct hospital costs and toxicity) will be assessed at baseline (week 0), at the end of O_3_/O_2_ insufflation (week 16), and at the end of follow-up (week 28).  **Primary Outcome Measures**   1. Change from baseline in "numbness and tingling" self-perceived by patients at the end of follow-up: Self-reported evaluation of the percentage of "numbness and/or tingling" regarding the basal level, from 100% (basal level, 0% improvement) to 0% (no numbness and tingling, 100% improvement), at the end of follow-up (week 28). 2. Change from baseline in HRQoL using the EQ-5D-5L questionnaire (developed by the EuroQol Group) self-perceived by patients at the end of follow-up: Self-reported evaluation of: a) 5 physical and emotional items scored in five levels, from 1 (Best: I have no problem) to 5 (worst: I have an extreme problem or I am unable to…) and b) additional self-assessment of health by a visual analogue scale (VAS) (0 = worst health patient can imagine, 100 = best health patient can imagine), at the end of follow-up (week 28).   **Secondary Outcome Measures**   1. Direct hospital costs: The direct expenses incurred by the hospital in providing services during the 28 weeks for the study (in euros). 2. Change from baseline in "numbness and tingling" self-perceived by patients at the end of O3T (week 16). 3. Change from baseline in HRQoL using the EQ-5D-5L questionnaire, self-perceived by patients at the end of O3T (week 16). 4. Changes from baseline in the Grade of toxicity of paraesthesia (numbness, tingling) according to the CTCAE v.5.0. scale (from the National Cancer Institute of EEUU). Range from Grade 0 (asymptomatic or mild symptoms) to Grade 3 (severe symptoms, limiting self-care activities in daily life). Assessed at the end of O3T (week 16) and at the end of follow-up (week 28). 5. Changes from baseline in the Grade of toxicity of sensory neuropathy according to the CTCAE v.5.0. scale. Range from Grade 0 (asymptomatic or mild symptoms) to Grade 4 (life-threatening consequences, urgent intervention indicated). Assessed at the end of O3T (week 16) and at the end of follow-up (week 28). 6. Changes from baseline in the degree of neuropathy according to the QLQ-CIPN20 scale from the European Organization for Research & Treatment in Cancer (EORTC). It is evaluated through 20 items grouped into 3 dimensions: sensitive, motor, and autonomic. Range: each item is scored from 1 (nothing) to 4 (a lot). The total score for each dimension is transformed into a score from 0 to 1000, with 0 being the best state and 100 the worst. Assessed at the end of O3T (week 16) and at the end of follow-up (week 28). 7. Changes from baseline in the HRQoL according to the QLQ-C30 questionnaire from the EORTC: Self-reported evaluation of 30 items that measure several scales and symptoms. Range (after standardization): from 0 (worst for overall health and function, best for symptoms) to 100 (best for overall health and functions, worst for symptoms). Assessed at the end of O3T (week 16) and at the end of follow-up (week 28). 8. Changes from baseline in levels of anxiety and depression according to the Hospital Anxiety and Depression Scale (HADS). HADS is a self-administered questionnaire that assesses 14 items/symptoms of anxiety (7) and depression (7) experienced by patients. Each item is scored from 0 (better, no alteration) to 3 (worse level of alteration). For each symptom (anxiety or depression), the overall score is from 0 (better, no anxiety or depression) to 21 (worse, very severe anxiety or depression). Assessed at the end of O3T (week 16) and at the end of follow-up (week 28). 9. Changes from baseline in biochemical parameters of oxidative stress (superoxide dismutase, glutathione, glutathione peroxidase, and free radicals). Assessed at the end of O3T (week 16) and at the end of follow-up (week 28). 10. Changes from baseline in biochemical parameters of inflammation cytokines. Assessed at the end of O3T (week 16) and at the end of follow-up (week 28). 11. Changes from baseline in hyperspectral signatures and infrared images obtained from hands and feet. Assessment of the percentage of reflectance for each wavelength of the hyperspectral and infrared images obtained with specific devices. Assessed at the end of O3T (week 16) and at the end of follow-up (week 28). 12. Toxicity of rectal O3T, recorded according to CTCAE v5.0. |
| Participant timeline | 13 | Time schedule of enrolment, interventions (including any run-ins and washouts), assessments, and visits for participants. A schematic diagram is highly recommended (see Figure 1) |
| Sample size | 14 | The sample size of 42 patients (21 per group) was calculated using GRANMO software (<https://www.datarus.eu/aplicaciones/granmo/>). This is based on an expected clinically relevant reduction in symptoms (>50% decrease in numbness and tingling from baseline) in at least 50% of the ozone group patients, compared to less than 10% in the placebo group, with 80% power, a one-sided alpha of 0.05, a 1:1 ratio, and accounting for 10% possible loss to follow-up. |
| Recruitment | 15 | Strategies for achieving adequate participant enrolment to reach the target sample size in a clinical trial: Engaging referring clinicians: Collaborate with oncologists, general practitioners, or other relevant specialists to identify and refer eligible patients. Patient outreach and education: Use flyers, informational sessions, social media, or community events to raise awareness about the study and its potential benefits. Providing clear informed consent materials: Ensure patients fully understand the study, its procedures, and potential benefits/risks, to increase willingness to participate. Leveraging patient registries or databases: Use hospital records, disease registries, or electronic medical records to identify potential participants systematically. Monitoring recruitment progress regularly: Track enrolment metrics weekly or monthly to identify bottlenecks early and adjust strategies as needed. |
| **Methods: Assignment of interventions (for controlled trials)** | | |
| Allocation: |  |  |
| Sequence generation | 16a | A stratified randomization will be carried out based on sex to ensure a homogeneous distribution between the two arms. Randomization will be managed by a statistician, who will be independent of those who analyse the results. Opaque, sealed envelopes containing the assigned treatment group (coded as Group A or Group B) will be used. The principal investigator will maintain a record of patient names, randomization dates, and code numbers. However, the key linking the code to the actual treatment (ozone or oxygen) will be known only to the randomization investigators and Chronic Pain Unit staff, remaining hidden until the analysis is completed. This ensures triple blinding of participants, care providers (oncologists/haematologists), and outcomes assessors. |
| Allocation concealment mechanism | 16b | Mechanism of implementing the allocation sequence: sequentially numbered: The allocation sequence will be implemented using sequentially numbered, sealed, opaque envelopes to ensure concealment until the point of assignment. |
| Implementation | 16c | Who will generate the allocation sequence: a statistician. who will enrol participants, and who will assign participants to interventions: principal investigator. |
| Blinding (masking) | 17a | Who will be blinded after assignment to interventions (eg, trial participants, care providers, outcome assessors, data analysts), and how: Opaque, sealed envelopes containing the assigned treatment group (coded as Group A or Group B) will be used. The principal investigator will maintain a record of patient names, randomization dates, and code numbers. However, the key linking the code to the actual treatment (ozone or oxygen) will be known only to the randomization investigators and Chronic Pain Unit staff, remaining hidden until the analysis is completed. |
|  | 17b | If blinded, circumstances under which unblinding is permissible, and procedure for revealing a participant’s allocated intervention during the trial: If unblinding is necessary, it will only be permitted in cases of medical emergencies where knowledge of the assigned intervention is essential for the participant's clinical management. In such cases, the principal investigator will unblind the treatment group. All unblinding events will be recorded and justified. |
| **Methods: Data collection, management, and analysis** | | |
| Data collection methods | 18a | Plans for assessment and collection of outcome, baseline, and other trial data, including any related processes to promote data quality (eg, duplicate measurements, training of assessors) and a description of study instruments (eg, questionnaires, laboratory tests) along with their reliability and validity, if known. Reference to where data collection forms can be found, if not in the protocol:  Outcome, baseline, and other trial data will be collected using standardized case report forms (CRFs) developed specifically for this study. Data collection will be performed by trained personnel following detailed standard operating procedures (SOPs) to ensure consistency and accuracy. Assessors will undergo specific training sessions to minimize inter-observer variability and promote data quality. When applicable, duplicate measurements will be conducted to verify reliability.  Patient-reported outcomes, including symptom severity and quality of life, will be assessed using validated instruments such as the Visual Analogue Scale (VAS) and the EORTC QLQ-CIPN20 questionnaire. Both tools have demonstrated good reliability and validity in oncology populations.  Laboratory tests and clinical assessments will follow standard hospital protocols and will be carried out in certified laboratories. |
|  | 18b | Plans to promote participant retention and complete follow-up, including list of any outcome data to be collected for participants who discontinue or deviate from intervention protocols: To promote participant retention and ensure complete follow-up, the study will implement several strategies, including regular contact with participants through phone calls, reminder messages, and flexible scheduling of visits to accommodate individual needs. Participants will be provided with clear information about the importance of follow-up regardless of adherence to the intervention protocol.  For participants who discontinue the intervention or deviate from the protocol, efforts will be made to collect key outcome data, including primary and secondary endpoints such as symptom severity (e.g., VAS scores), quality of life measures (e.g., EORTC QLQ-CIPN20), and any reported adverse events. These participants will continue to be included in the intention-to-treat analysis to preserve the integrity of the study findings. |
| Data management | 19 | Plans for data entry, coding, security, and storage, including any related processes to promote data quality (eg, double data entry; range checks for data values). Reference to where details of data management procedures can be found, if not in the protocol:  Data will be entered into a secure, password-protected electronic database by trained personnel. To ensure data quality, the study will implement double data entry for critical variables and perform regular range and consistency checks to identify and correct discrepancies. All data will be coded using standardized coding systems, and personal identifiers will be removed or anonymized to protect participant confidentiality.  Access to the database will be restricted to authorized study staff only. Regular backups will be conducted, and data will be stored on encrypted servers in compliance with institutional and regulatory requirements.  If not fully detailed in the study protocol, additional information on data management procedures will be available in the study’s Data Management Plan (DMP), which can be provided upon request. |
| Statistical methods | 20a | Statistical methods for analysing primary and secondary outcomes. Reference to where other details of the statistical analysis plan can be found, if not in the protocol:  A double analysis will be performed: "by intention to treat" (all included patients) and "by protocol" (only patients who completed the study in their assigned group). Normality will be assessed using the Kolmogorov-Smirnov test. Depending on data distribution, parametric or non-parametric tests will be used for comparisons between groups. Contingency tables will use the chi-square test. Variables with more than two follow-up assessments will be analysed using a mixed generalized linear model with repeated measures. Bonferroni adjustment will be applied for multiple comparisons, with a p-value < 0.05 considered significant. |
|  | 20b | Methods for any additional analyses (eg, subgroup and adjusted analyses): non applicable. |
|  | 20c | Definition of analysis population relating to protocol non-adherence (eg, as randomised analysis), and any statistical methods to handle missing data (eg, multiple imputation): The primary analysis will follow the intention-to-treat (ITT) principle, including all participants as randomized, regardless of protocol adherence. This approach preserves the benefits of randomization and provides an unbiased estimate of treatment effect.  For participants who deviate from the protocol or discontinue the intervention, all available outcome data will be included in the analysis. To handle missing data, we will use appropriate statistical methods such as multiple imputation, assuming data are missing at random. Sensitivity analyses may also be conducted to assess the impact of different missing data assumptions on the results. |
| **Methods: Monitoring** | | |
| Data monitoring | 21a | Composition of data monitoring committee (DMC); summary of its role and reporting structure; statement of whether it is independent from the sponsor and competing interests; and reference to where further details about its charter can be found, if not in the protocol. Alternatively, an explanation of why a DMC is not needed.  A Data Monitoring Committee (DMC) is not deemed necessary for this study due to its relatively low-risk nature, the non-invasive intervention (rectal ozone therapy), and the short to moderate duration of participant involvement. The study does not involve high-risk treatments or vulnerable populations, and the expected adverse events are minimal and well-characterized based on prior clinical experience.  Safety will be monitored internally by the principal investigator and the clinical research team, who will review adverse events and protocol adherence on an ongoing basis. In case of any serious or unexpected adverse events, these will be reported immediately to the ethics committee and relevant regulatory authorities, as per standard procedures.  Given these factors, the establishment of an independent DMC is not required. Full details of the safety monitoring procedures are included in the study protocol. |
|  | 21b | Description of any interim analyses and stopping guidelines, including who will have access to these interim results and make the final decision to terminate the trial:  An interim safety analysis will be conducted when patient #21 completes follow-up, with the possibility of premature study termination if unforeseen serious complications or differences are statistically and clinically relevant in the main clinical outcomes after evaluation by the Ethics Committee  Safety and participant well-being will be continuously monitored by the principal investigator and the clinical research team. In the event of unexpected adverse events or safety concerns, the research team will review the data and, if necessary, consult the ethics committee to determine whether the trial should be modified or terminated. Access to any such safety data will be restricted to the research team responsible for monitoring adverse events. |
| Harms | 22 | Plans for collecting, assessing, reporting, and managing solicited and spontaneously reported adverse events and other unintended effects of trial interventions or trial conduct: All adverse events (AEs), whether solicited or spontaneously reported, will be systematically collected throughout the study period. Participants will be asked about any symptoms or changes in health during each scheduled visit, and any reported AEs will be documented in the case report forms (CRFs).  Each AE will be assessed by the clinical research team in terms of severity, duration, and relationship to the study intervention. Serious adverse events (SAEs) will be reported immediately to the ethics committee and relevant regulatory authorities, in accordance with applicable guidelines.  All AEs and SAEs will be managed according to standard clinical practice. Appropriate medical care will be provided as needed, and participants will be followed until the resolution or stabilization of the event. The principal investigator will be responsible for ensuring that all safety information is reviewed regularly and that appropriate action is taken if safety concerns arise. |
| Auditing | 23 | Frequency and procedures for auditing trial conduct, if any, and whether the process will be independent from investigators and the sponsor:  No formal auditing is planned for this trial due to its low-risk nature and limited scale. However, internal monitoring procedures will be implemented to ensure compliance with the protocol, Good Clinical Practice (GCP) guidelines, and regulatory requirements.  Monitoring activities will be carried out by designated study personnel who are not involved in patient recruitment or intervention delivery, to maintain a degree of independence. These procedures will include periodic review of consent forms, data entry accuracy, and adverse event reporting.  If requested by regulatory authorities or the ethics committee, additional independent audits may be conducted. Any such audits would be carried out by individuals or organizations independent from the investigators and the sponsor. |
| Ethics and dissemination | | |
| Research ethics approval | 24 | Plans for seeking research ethics committee/institutional review board (REC/IRB) approval  The study protocol has already received approval from the appropriate Research Ethics Committee/Institutional Review Board (REC/IRB). Full details of the ethical approval, including the name of the committee, approval number, and date of approval, are provided in the Declarations section of the manuscript. Any protocol amendments will be submitted for further ethical review as required. |
| Protocol amendments | 25 | Plans for communicating important protocol modifications (eg, changes to eligibility criteria, outcomes, analyses) to relevant parties (eg, investigators, REC/IRBs, trial participants, trial registries, journals, regulators)  Any important protocol modifications—such as changes to eligibility criteria, outcomes, or statistical analyses—will be promptly communicated to all relevant parties. These include the investigators, the Research Ethics Committee/Institutional Review Board (REC/IRB), trial participants (when applicable), the trial registry, and, if necessary, regulatory authorities and journals.  All amendments will be documented clearly, and updates will be made in the trial registry entry. Investigators will be informed through official written communication, and participants will be re-consented if the changes affect their participation or safety. |
| Consent or assent | 26a | Who will obtain informed consent or assent from potential trial participants or authorised surrogates, and how (see Item 32)  Informed consent will be obtained by trained members of the clinical research team, specifically designated by the principal investigator. The process will take place in a private setting, ensuring adequate time and opportunity for potential participants to ask questions and fully understand the study objectives, procedures, risks, and benefits.  Consent will be obtained in writing before any study-related procedures are initiated. If a participant is unable to provide consent directly, an authorised legal representative or surrogate may do so, in accordance with local regulations and ethical guidelines. All consent procedures will follow Good Clinical Practice (GCP) standards and be documented appropriately. |
|  | 26b | Additional consent provisions for collection and use of participant data and biological specimens in ancillary studies, if applicable: Separate informed consent forms will be used for the collection of biological samples related to this study. |
| Confidentiality | 27 | How personal information about potential and enrolled participants will be collected, shared, and maintained in order to protect confidentiality before, during, and after the trial:  Personal information about potential and enrolled participants will be collected using secure, standardized case report forms and stored in a password-protected electronic database with restricted access. Each participant will be assigned a unique study identification code to anonymize data; identifiable information will be stored separately from clinical data.  Data sharing will be limited to authorized study personnel and, when required, ethics committees or regulatory authorities, always in compliance with data protection regulations. During the trial, all data handling will follow Good Clinical Practice (GCP) and institutional policies to ensure confidentiality. After the trial, data will be securely archived for the period required by law and institutional guidelines, and then disposed of or anonymized appropriately. |
| Declaration of interests | 28 | Financial and other competing interests for principal investigators for the overall trial and each study site:  Several approved O3T devices will be used interchangeably in this study. One of them (Ozonosan Alpha-plus®) was provided by Hänsler Medical GmbH, Iffezheim, Germany). Three other devices (Ozonobaric-P, SEDECAL, Madrid, Spain) were supported by a grant (COV20/00702) from the Instituto de Salud Carlos III (Spanish Ministry of Science and Innovation, Madrid, Spain).  In 2023, B.C. received financial support for a European grant application from Hänsler Medical GmbH (Iffezheim, Germany).  The authors declare that the research will be conducted without any commercial or financial relationships that could be construed as a potential conflict of interest. The authors declare that the funders had no role in the design of the study, in the collection, analysis, or interpretation of data, nor in the writing of this protocol, nor in the decision to publish the results, nor in the final manuscript. The authors declare no other potential conflict of interest than those described above. |
| Access to data | 29 | Statement of who will have access to the final trial dataset, and disclosure of contractual agreements that limit such access for investigators: Access to the final trial dataset will be limited to the principal investigator and designated members of the research team directly involved in data analysis. All individuals with access will be bound by confidentiality agreements and data protection policies.  There are no contractual agreements or restrictions that limit access to the dataset for the investigators. The study team retains full control over the data and its analysis, interpretation, and publication. |
| Ancillary and post-trial care | 30 | Provisions, if any, for ancillary and post-trial care, and for compensation to those who suffer harm from trial participation:  Participants who experience any harm related to trial participation will receive appropriate medical care in accordance with institutional and national regulations. The study sponsor has arranged insurance coverage to provide compensation for any trial-related injury, as required by local laws.  Although no specific ancillary or post-trial care is planned due to the low-risk nature of the intervention, participants will continue to receive their standard medical care as prescribed by their treating physicians. Any medical issues arising during or after the trial will be managed following standard clinical practice. |
| Dissemination policy | 31a | Plans for investigators and sponsor to communicate trial results to participants, healthcare professionals, the public, and other relevant groups (eg, via publication, reporting in results databases, or other data sharing arrangements), including any publication restrictions:  The trial results will be communicated to participants, healthcare professionals, and the public through multiple channels. A summary of the study findings will be provided to participants in a clear and accessible format after the trial’s completion. Results will also be submitted for publication in peer-reviewed scientific journals and presented at relevant conferences.  Additionally, the trial outcomes will be reported in recognized clinical trial registries in accordance with regulatory requirements. Data sharing will comply with ethical standards and participant confidentiality.  There are no publication restrictions imposed by the sponsor; investigators retain full rights to publish and disseminate the study results. |
|  | 31b | Authorship eligibility guidelines and any intended use of professional writers: Authorship eligibility will follow the International Committee of Medical Journal Editors (ICMJE) criteria, requiring substantial contributions to the study design, data acquisition, analysis, or interpretation; drafting or revising the manuscript critically for important intellectual content; final approval of the version to be published; and agreement to be accountable for all aspects of the work.  All individuals meeting these criteria will be offered authorship. Contributions that do not meet authorship criteria will be acknowledged appropriately.  Professional medical writers may be engaged to assist with manuscript preparation, but their involvement will be fully disclosed in the publication, and they will not influence the study design, data analysis, or interpretation. |
|  | 31c | Plans, if any, for granting public access to the full protocol, participant-level dataset, and statistical code:  The full study protocol will be made publicly available by publishing it as a supplementary file alongside the main manuscript in the chosen journal (BMC Cancer). This ensures transparency and allows other researchers to review the study design in detail.  Participant-level datasets and statistical code will be shared upon reasonable request, subject to approval by the principal investigator and compliance with data protection regulations to safeguard participant confidentiality. Data sharing agreements will be established to ensure appropriate use.  There are currently no plans for unrestricted public access to participant-level data or code, but efforts will be made to facilitate data sharing with qualified researchers to promote scientific collaboration. |
| Appendices |  |  |
| Informed consent materials | 32 | Model consent form and other related documentation given to participants and authorised surrogates. Attached |
| Biological specimens | 33 | Plans for collection, laboratory evaluation, and storage of biological specimens for genetic or molecular analysis in the current trial and for future use in ancillary studies, if applicable:  Specific informed consent forms will be obtained separately for the collection and storage of biological samples in this study. These consent forms were also approved by the Ethics Committee. All samples will be handled, stored, and used in accordance with applicable regulations and guidelines to ensure confidentiality and participant safety. |

Figure 1. Time Schedule of Enrolment, Interventions, Assessments, and Visits

| **Week** | **0** | **1 - 8** | **9 - 16** | **17 - 28**  **(Follow-up)** |
| --- | --- | --- | --- | --- |
| **Enrolment** | Screening, Baseline assessments |  |  |  |
| **Interventions** |  | Rectal insufflation: 3 sessions/week (total 24 sessions) | Rectal insufflation: 2 sessions/week (total 16 sessions) | No experimental intervention (follow-up only) |
|  |  | Ozone Group: O_3_/O_2_ gas, increasing concentration (10→30 µg/mL) | Ozone Group: O_3_/O_2_ gas at 30 µg/mL |  |
|  |  | Control Group: O_2_ only (0 µg/mL) | Control Group: O_2_ only (0 µg/mL) |  |
| **Procedure Details** |  | Rectal probe insertion, volume 180-300 mL if tolerated | Same as weeks 1-8 |  |
| **Assessments** | Baseline: Numbness/tingling, HRQoL (EQ-5D-5L), biochemical, imaging, toxicity scales | End of treatment (week 16): Primary and Secondary outcomes including: - Numbness/tingling change - HRQoL EQ-5D-5L and QLQ-C30 - Toxicity grades (CTCAE v5.0) - Neuropathy QLQ-CIPN20 - HADS - Biochemical parameters (oxidative stress, cytokines) - Hyperspectral and infrared imaging |  | End of follow-up Re-assessment of  Primary and Secondary outcomes: - Same as basal (week 0). Additionally:  - Hospital costs  - Rectal O3T toxicity |
| **Follow-up Visits** |  |  |  | Regular follow-up visits for 12 weeks post-treatment |

**Notes:**

Key assessments at **Week 0 (Baseline), Week 16 (end of intervention)**, and **Week 28 (end of follow-up).** Secondary variables include hospital costs, toxicity, quality of life, biochemical parameters, neuropathy signs, mental health, and imaging analyses. Total patient duration: 28 weeks. Total project duration: 60 months.

**Legend:**

**CTCAE:** Common Terminology Criteria for Adverse Events; **EORTC:** European Organization for Research and Treatment of Cancer; **HADS:** Hospital Anxiety and Depression Scale; **HRQoL:** Health-Related Quality of Life; **O3T:** Ozone Treatment; **QLQ-C30:** Quality of Life Questionnaire-Core 30; **QLQ-CIPN20:** Quality of Life Questionnaire-Chemotherapy-Induced Peripheral Neuropathy 20-item scale; **VAS:** Visual Analog Scale.
